# Sodium-Glucose Cotransporter-2 Inhibitors-from the Treatment of Diabetes to Therapy of Chronic Heart Failure

**DOI:** 10.3390/jcdd9070225

**Published:** 2022-07-14

**Authors:** Dominik Kurczyński, Bartosz Hudzik, Marta Jagosz, Jan Zabierowski, Jolanta Nowak, Andrzej Tomasik, Arkadiusz Badziński, Piotr Rozentryt, Mariusz Gąsior

**Affiliations:** 1 2nd Department of Cardiology, Faculty of Medical Sciences in Zabrze, Medical University of Silesia, 10 Curie-Sklodowska Str., 41-808 Zabrze, Poland; tomasik@poczta.onet.pl; 2 Department of Cardiovascular Disease Prevention, Faculty of Health Sciences in Bytom, Medical University of Silesia, Piekarska 18 Street, 41-902 Bytom, Poland; bartekh@mp.pl; 3 Silesian Center for Heart Diseases, Third Department of Cardiology, Faculty of Medical Science in Zabrze, Medical University of Silesia, 41-800 Zabrze, Poland; nowjola@wp.pl (J.N.); prozentryt@sum.edu.pl (P.R.); m.gasior@sccs.pl (M.G.); 4 Faculty of Medical Sciences in Zabrze, Medical University of Silesia, 40-055 Katowice, Poland; martajagosz96@gmail.com (M.J.); kancelaria@badzinski.com.pl (A.B.); 5 Department of Cardiology, Faculty of Medical Sciences in Zabrze, Silesian Centre for Heart Disease, Medical University of Silesia, 41-800 Zabrze, Poland; janzabierowski@poczta.onet.pl; 6 Silesian Nanomicroscopy Center, Silesia LabMed: Research and Implementation Center, Medical University of Silesia, 41-800 Zabrze, Poland; 7 Department of Toxicology and Health Protection, Faculty of Health Sciences in Bytom, Medical University of Silesia in Katowice, 41-902 Bytom, Poland

**Keywords:** diabetes, gliflozins, heart failure, SGLT2, sodium-glucose cotransporter-2 inhibitors

## Abstract

Sodium-glucose cotransporter-2 (SGLT2) inhibitors are currently the second-line pharmacotherapy in type 2 diabetes, particularly through their effectiveness in reducing glycemia, but also due to their cardioprotective and nephroprotective effects. In light of surprisingly satisfactory results from large, randomized trials on gliflozins, SGLT2 received the highest recommendation (Class IA) with the highest level of evidence (A) in the treatment algorithm for HF with reduced LVEF in recent ESC HF guidelines. This great breakthrough in the treatment of HF is due to different mechanisms of action of gliflozins that are reported to be able to change the natural course of HF by reducing the risk of both hospitalization and death. They are recommended regardless of the patient’s diabetes status. This review summarizes the up-to-date literature on their beneficial and pleiotropic impact on the cardiovascular system.

## 1. Introduction

Sodium-glucose cotransporter-2 (SGLT2) inhibitors have a long history, which began with the isolation of phlorizin from apple tree bark and hence the name of the group (gliflozins). They were initially used as antipyretics. Later, sodium-glucose transporters were discovered. Animal studies confirmed the ability of these substances to reduce blood glucose levels and increase tissue sensitivity to insulin. As a result of further research, dapagliflozin, a selective inhibitor of renal SGLT2 transporter, was the first drug from this group to be introduced to the treatment of diabetes in 2012 [1,2,3,4,5]. The mechanism of action of gliflozins, which is based on the inhibition of renal glucose reabsorption and increased glucosuria, also leads to reduced glycemia, increased energy loss, diuresis, and natriuresis. These drugs initially had an established position in the treatment of diabetes. However, currently, they are becoming one of the mainstay treatments for patients with chronic heart failure with reduced left ventricular ejection fraction (HFrEF) [6]. Studies have confirmed their highly beneficial effects on the cardiovascular system and their cardio- and renal-protective effects [7,8,9].

## 2. SGLT2 Inhibitors in the Treatment of Type 2 Diabetes

According to the most recent joint recommendations of the American Diabetes Association (ADA) and the European Association for the Study of Diabetes (EASD) published in 2018 and updated in 2019, the choice of pharmacotherapy in type 2 diabetes depends mainly on the comorbidity of cardiovascular disease and chronic kidney disease [10]. The first choice drug is still metformin, whose action was even more strongly emphasized. SGLT2 inhibitors, primarily recommended for obese patients at high risk of hypoglycemia who either cannot tolerate metformin or require combination therapy, became second-line drugs primarily due to their cardioprotective and nephroprotective effects. The introduction of SGLT2 inhibitors into the recommendations resulted from many studies that confirmed their efficacy in reducing glycemia in monotherapy [11,12] or combination with metformin [13,14].

The results of the cardiovascular safety of gliflozins were surprising (Table 1). In three large, randomized trials conducted on the population with diagnosed or at high risk of cardiovascular disease, the use of SGLT2 inhibitors was associated with a significant reduction in the risk of adverse cardiovascular events. In addition, an exceptionally substantial reduction was found in the rate of hospitalization for heart failure (HF) [8,15,16].

## 3. SGLT2 Inhibitors in the Treatment of Heart Failure

There has been a breakthrough in the treatment of chronic HFrEF. The recent introduction of SGLT2 inhibitors to the treatment of patients with HFrEF with and without diabetes is a milestone for effective HF treatment and improved prognosis in this patient group [17]. The use of SGLT2 inhibitors in patients with type 2 diabetes allowed for effective secondary prevention of cardiovascular disease and particularly for the prevention of hospitalization for HF in patients with type 2 diabetes (DECLARE-TIMI 58 and EMPA-REG OUTCOME trials) [8,15].

Moreover, the CREDENCE trial showed that these drugs inhibited the progression of chronic kidney disease [18]. Such favorable results of SGLT2 inhibitors concerning cardiovascular disease resulted in the changes in the European Society of Cardiology guidelines developed in collaboration with the European Society for the Study of Diabetes. These changes were related to diabetes and the pre-diabetic condition coexisting with cardiovascular disease [19]. Therefore, empagliflozin, canagliflozin, and dapagliflozin in type 2 diabetes patients with a high risk for HF are a Class IA recommendation due to their high efficacy in preventing hospitalization for HF. However, the authors of the guidelines could not explain how SGLT2 inhibitors improved HF outcomes or whether these drugs also reduced HF risk in patients without diabetes. The DAPA-HF trial partially explained the above phenomena. HFrEF patients with and without diabetes were enrolled in the study. The DAPA-HF trial included 4744 patients with left ventricular ejection fraction (LVEF) < 40% (mean EF 31%), II-IV New York Heart Association (NYHA) class who were treated according to the current recommendations.

Most of them were given diuretics. Angiotensin-converting enzyme inhibitors (ACEIs) and angiotensin II receptor antagonists (ARBs) were given to 94% of patients, 96% of subjects were treated with beta-blockers, and 71% of patients were given a mineralocorticoid receptor antagonists. Only 10% of patients were administered an ARB in combination with a neprilysin inhibitor (ARNI). 55% of patients with HFrEF were not diagnosed with type 2 diabetes and were randomly assigned to two groups. The first group was given dapagliflozin (10 mg), while the other group received placebo. After 18 months, a 26% reduction in the incidence of the primary endpoint of cardiovascular death, hospitalization for HF, or emergency visit related to HFrEF exacerbation which required intravenous drug therapy, was reported in the dapagliflozin group (HR 0.74; 95% CI 0.65–0.85; *p* < 0.001). Additionally, lower overall mortality was found in the dapagliflozin group (11.6% vs. 13.9% HR 0.83; 95% CI 0.71–0.97). Interestingly, the same beneficial effect of dapagliflozin was reported in patients with and without diabetes. The drug had no significant effect on glycated hemoglobin levels in HFrEF patients without diabetes, nor did it cause the risk of hypoglycemia. The use of the drug prevented hospitalization, worsening HF, or causing death in one of 21 patients [20]. This finding is comparable to the results achieved by using ARNIs, as reported in the PARADIGM-HF trial [21]. A DAPA-HF sub-analysis showed that the benefits of dapagliflozin were similar in patients treated with ARNIs and in those who were not treated with these drugs [22].

The EMPEROR-Reduced trial was another research that demonstrated the efficacy of gliflozins in the treatment of HFrEF. The trial included 3730 patients with LVEF ≤ 40% (NYHA II-III) optimally treated with pharmacological agents. Drugs inhibiting the renin-angiotensin system were used by 70% of patients, while 18% of subjects were treated with ARNIs. Beta-blockers were taken by 94%, while aldosterone antagonists by 70% of patients. Compared with the DAPA-HF trial, the participants of the EMPEROR-Reduced trial had lower baseline LVEF, were more often treated with ARNIs, and had higher NT-proBNP levels (Table 2). At 16-month follow-up, the use of empagliflozin was associated with a lower risk of cardiovascular death or HF hospitalization compared with placebo (HR 0.75; 95% CI 0.65–0.86; *p* < 0.001). This difference was mainly due to a lower incidence of HF hospitalization in the empagliflozin group (HR 0.69; 95% CI 0.59–0.81; *p* < 0.001) [23]. Empagliflozin was also effective for secondary endpoints, which resulted in a 30% reduction in total HF hospitalizations (388 vs. 553 hospitalizations, HR 0.70; 95% CI 0.58–0.85; *p* < 0.001) and a slower decline in glomerular filtration rate (GFR) compared with placebo (−0.55 vs. −2.28 mL/min/1.73 m^2^ per year, HR 1.73; 95% CI 1.1–2.37; *p* < 0.001). The benefits were irrespective of type 2 diabetes, age, HF etiology, or the treatment.

In the EMPEROR-Reduced and DAPA-HF trials, the reduction in the risk of adverse cardiovascular events in HFrEF patients with and without type 2 diabetes was mainly associated with a decrease in HF hospitalization rate. However, as opposed to dapagliflozin, which reduced the overall mortality by 17% in the DAPA-HF trial (HR 0.83; 95% CI 0.71–0.97), empagliflozin did not reduce the risk of all-cause death and reduced the risk of cardiovascular death by 8% only compared with the control group in the EMPEROR-reduced trial (HR 0.92; 95% CI 0.75–1.12). On the other hand, dapagliflozin reduced the risk of cardiovascular death by 18% (HR 0.82; 95% CI 0.69–0.98).

It is difficult to interpret these data unequivocally since the results may have been influenced by biased study groups: a more advanced stage of HFrEF in patients from the EMPEROR-Reduced trial (Table 2), and the fact that death was not a prespecified endpoint in both studies. However, the benefits of these drugs in patients with HFrEF were evident in both trials, and the reduction in the adopted primary endpoints was similar in both studies. A meta-analysis of DAPA-HF and EMPEROR-Reduced trials showed that the use of an SGLT2 inhibitor in patients with HFrEF resulted in a 13% reduction in total mortality (pooled HR 0.87, 95% CI 0.77–0.98; *p* = 0.018), a 14% reduction in cardiovascular mortality (HR 0.86, 95% CI 0.76–0.98; *p* = 0.027) [25] and a 38% reduction in adverse renal events (HR 0.62, 95% CI 0.43–0.90; *p* = 0.013). The beneficial effects of dapagliflozin and empagliflozin were not related to age, sex, diabetes, ARNI treatment, or baseline GFR.

The efficacy of sotagliflozin in the treatment of HF was assessed in the SOLOIST-WHF trial. As opposed to dapagliflozin and empagliflozin, sotagliflozin is an inhibitor of SGLT1 and SGLT2. SGLT1 is mainly located in the intestines, and its inhibition is associated with delayed glucose absorption [26]. In the SOLOIST-WHF trial, the study group included patients with heart failure with either reduced or preserved left ventricular ejection fraction (HFpEF) and type 2 diabetes (Table 2). Patients were enrolled in the study during hospitalization for decompensated HF or within three days after hospital discharge. The study was terminated prematurely due to a lack of further funding. Compared with placebo, sotagliflozin contributed to a significant reduction in the incidence of the composite endpoint defined as death from cardiovascular causes and hospitalization or emergency visit for HF. There were 51 adverse events per 100 patient-years in the sotagliflozin group, while 76.3 adverse events per 100 patient years were reported in the placebo group (HR 0.67, 95% CI 0.52–0.85; *p* < 0.001) [24]. Sotagliflozin did not reduce death rates from cardiovascular or any cause. However, hypoglycemia and diarrhea were more prevalent in the treatment arm. The SOLOIST-WHF trial demonstrated the benefits of the early use of sotagliflozin in patients with type 2 diabetes and HF after an episode of acute HF. Unfortunately, because of the relatively small number of patients with HFpEF and the early trial termination, the authors could not answer whether patients with HFpEF could have the same beneficial effect from treatment with SGLT2 inhibitors as patients with HFrEF.

The EMPEROR-Preserved trial was the first research on the efficacy of SGLT2 inhibitors in the treatment of HFpEF. The trial included 5988 patients with HF and LVEF > 40%. Compared with placebo, treatment with empagliflozin (10 mg/day was associated with a significant reduction in the combined endpoint of cardiovascular death and HF hospitalization (13.8% vs. 17.1% HR 0.79, 95% CI 0.69–0.90, *p* < 0.001) [27].

As in EMPEROR-Reduced and DAPA-HF trials, the benefits of the SGLT2 inhibitor were consistent across patients with and without diabetes. The analysis of the EMPEROR-Preserved trial results adjusted to LVEF showed a similar reduction in adverse cardiovascular events in the group with LVEF > 50% and the group with LVEF between 40% and 49%. Treatment with empagliflozin in this group of patients was also associated with a significant improvement in the quality of life as measured by the Kansas City Cardiomyopathy Questionnaire. The results of the EMPEROR-Preserved trial indicated that empagliflozin was one of the few drugs effective in HF with reduced and preserved ejection fraction.

## 4. Cardioprotective Effects of SGLT2 Inhibitors

**Figure 1:**
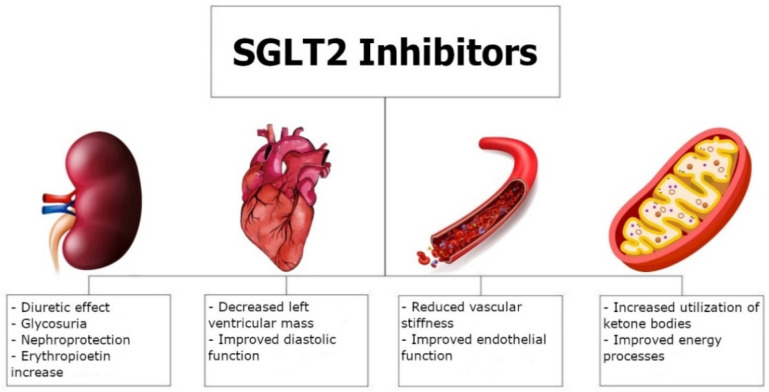
Pleiotropic mechanism of action of SGLT2 inhibitors.

The mechanism by which SGLT2 inhibitors exert such a potent cardioprotective effect in HFrEF patients without diabetes remains unclear. Dapagliflozin shows a pleiotropic activity that seems to be common to the entire group of SGLT2 inhibitors (Figure 1). Beneficial cardiovascular effects of gliflozins seem to be related to a reduction in blood pressure resulting from a diuretic effect, a reduction in arterial stiffness, and a decrease in sympathetic nervous system activity [28,29,30]. Improved outcomes in HFrEF patients may also result from improved myocardial bioenergetics and structure, as well as increased erythropoietin production [31,32].

### 4.1. Diuretic Effect

The early hemodynamic benefits of lowering blood pressure in patients on SGLT2 inhibitors may be related to osmotic diuresis and natriuresis. The diuretic effect of SGLT2 inhibitors is a direct result of the inhibition of SGLT2 in the kidney proximal tubule. Under physiological conditions, SGLT2 is responsible for glucose reabsorption and approximately 5% of filtered sodium. However, under hyperglycemic conditions, the reabsorption of glucose and sodium is increased. Thus, inhibition of the SGLT2 receptor induces natriuresis and osmotic diuresis [28]. Many studies on patients with type 2 diabetes found an increased volume of urine output during the initial phase of treatment [2,33]. However, increased diuresis is a transient phenomenon. In one study, urine production returned to baseline within four weeks of treatment [34]. In patients with HF, diuresis induced by gliflozins may also result from interactions with the sodium-hydrogen exchanger (NHE3), mainly located in the kidneys. NHE3 is an essential protein responsible for most sodium reabsorption. This protein may be involved in the pathogenesis of fluid retention and peripheral edema formation and resistance to endogenous natriuretic peptides or diuretics [35]. Increased activity of NHE1 in the cardiovascular system and NHE3 play a crucial role in the pathophysiology of HF and diabetes [36]. Gliflozins reduce the activity of this protein in an unclear mechanism [37].

SGLT2 inhibitors show significant differences in their effect on water metabolism compared to classical diuretics. Hallow et al. studied the diuretic effect of dapagliflozin and bumetanide in healthy volunteers. They indicated that dapagliflozin reduced more extravascular water volume than intravascular water volume. This phenomenon may help control peripheral edema without adverse effects on arterial perfusion in patients with HF [38].

### 4.2. Reduction in Vascular Stiffness and Sympathetic Nervous System Activity

The reduction in systolic blood pressure during treatment with gliflozins is due to a diuretic effect and a decrease in afterload due to reduced arterial stiffness [39]. Bosch et al., in their sub-analysis focusing on the mechanisms responsible for this effect, highlighted the anti-inflammatory properties of empagliflozin leading to improved endothelial function [29]. Another hypothesis is that the sympathetic nervous system activity is reduced when SGLT2 inhibitors are used [40]. Excessive sympathetic activation plays a vital role in the development of hypertension and HF. The hypothesis of reduced sympathetic nervous system activity is based on the observation of patients with type 2 diabetes on empagliflozin, who did not present with increased heart rate despite a decrease in blood pressure [30]. Moreover, other authors did not observe reflex activation of the sympathetic nervous system during treatment with gliflozins, which is characteristic of diuretics [40,41]. Animal studies also support the hypothesis of reduced sympathetic nervous system activity. Mice administered dapagliflozin showed reduced blood pressure and decreased cardiac and renal tyrosine hydroxylase and norepinephrine levels, which are markers of sympathetic nervous system activity [42].

### 4.3. Impact of SGLT2 Inhibitors on the Myocardium

A series of studies on animal models showed beneficial effects of SGLT2 inhibitors on the myocardium. Younis et al. reported a reduction in blood pressure, mass, and left ventricular dilatation on echocardiography in rats treated with empagliflozin compared to the placebo group [43]. Habibi et al., in a group of mice with obesity and diabetes treated with empagliflozin, found an improvement in left ventricular diastolic function defined as a decrease in E/e’ (corresponding to left ventricular late diastolic pressure) and favorable morphological changes in the myocardium, including a reduction in fibrosis, cardiomyocyte hypertrophy and changes in the mitochondrial cellular level despite no significant reduction in blood pressure [44]. Similar results were obtained in patients with type 2 diabetes using SGLT2 inhibitors. The EMPA-Heart trial demonstrated a significant reduction in the left ventricular mass in 97 normotensive patients with diabetes and chronic coronary syndrome without HF. After six months of empagliflozin treatment, the left ventricular mass index assessed by magnetic resonance (MR) was reduced on average by 2.6 g/m^2^ compared with a reduction of 0.01 g/m^2^ in the placebo group (adjusted difference −3.35 g/m^2^; 95% CI −5.9 to −0.81 g/m^2^, *p* = 0.01). At the same time, systolic blood pressure (adjusted difference −6.8 mmHg, 95% CI −11.2 to −2.3 mmHg, *p* = 0.003) and diastolic blood pressure (adjusted difference −3.2 mmHg, 95% CI −5.8 to −0.6 mmHg, *p* = 0.02) were reduced in the empagliflozin group compared to placebo [45]. Left ventricular ejection fraction (LVEF) increased slightly from 58.0 to 59.1% (adjusted difference 2.2%, 95% CI −0.2 to 4.7%, *p* = 0.08). Several years earlier, after three months of treatment with empagliflozin, the same group of investigators reported reduced mean left ventricular mass index from 88.2 to 75.4 g/m^2^ (*p* = 0.01) and improved diastolic function per the early lateral annular tissue Doppler velocity (lateral e’) from 8.5 to 9.7 cm/s (*p* = 0.002) in 10 patients with type 2 diabetes and cardiovascular disease. Those investigators found no significant changes in LVEF or left ventricular volume [45]. Similarly, after three months of treatment with canagliflozin, 37 patients with type 2 diabetes, hyperlipidemia mostly accompanied by hypertension and cardiovascular disease (32.4%) showed a significant reduction in the E/e’ ratio (mitral annular septal area) from 13.7 ± 3.5 to 12.1 ± 2.8 (*p* = 0.001) [46].

In turn, six months after the administration of dapagliflozin, in the study by Soga et al., E/e’ showed a significant decrease from 9.3 to 8.5 cm/s (*p* = 0.020). At the same time, they showed a significant reduction in the left atrial volume index (LAVI) from 31 to 26 mL/m^2^ (*p* = 0.001) and the left ventricular mass index (LVMI) from 75.0 to 67.0 g/m^2^ (*p* < 0.001) [47]. The exact mechanism by which SGLT2 inhibitors improve diastolic function and reduce the left ventricular mass is unclear. Presumably, by increasing sodium and glucose excretion, they reduce the intravascular volume, which corresponds to a decrease in the preload and afterload of the myocardium, more efficient myocardial function, and reduced oxygen consumption [7]. Combined with their effect on arterial stiffness, it may reduce left ventricular mass and improve diastolic function [45].

SGLT2 inhibitors may interfere with myocardial metabolism [48]. By inhibiting the SGLT2 receptor, they induce glucosuria and decrease blood glucose and insulin levels, which is related to an increase in glucagon concentration. The above changes lead to increased ketone body production in the liver. Patients treated with SGLT2 inhibitors showed an approximately twofold increase in blood ketone bodies [46]. During ketonemia, various organs take beta-hydroxybutyric acid (mainly the heart muscle), thus displacing the oxidation of free fatty acids. In addition, hemoconcentration that occurs when SGLT2 receptors are blocked increases the release of oxygen to cells which, together with a shift in metabolic substrates, increases the beneficial effect of SGLT2 inhibitors [46].

This appears to be particularly important in the failing heart, in which adverse changes in cardiomyocyte metabolism are reported. As HF progresses, the activity of enzymes involved in mitochondrial fatty acid oxidation decreases. This process in cardiomyocytes is initially accompanied by an increase in glucose uptake and glycolysis. However, over time, insulin resistance develops in the failing heart, and glucose uptake decreases. This process may lead to a decrease in adenosine triphosphate (ATP) production and progression of HF [31]. Studies have suggested increased utilization of ketone bodies by the failing myocardium, which could provide an alternative energy source for the damaged myocardium [49,50,51]. Increased availability of ketone bodies corresponds to increased ATP production [52].

Increased expression of enzymes involved in the metabolism of ketone bodies is reported in cardiomyocytes of the failing heart. Compared to free fatty acids, ketone bodies have a higher energy efficiency expressed as the phosphate-to-oxygen ratio (ATP per reduced oxygen atom) [31,53]. There are indications that intravenous infusion of 3-hydroxybutyric acid, the predominant ketone body in HF, leads to beneficial hemodynamic effects such as increased cardiac output and LVEF [54]. Additionally, beta-hydroxybutyric acid has anti-inflammatory properties by blocking the NLRP3 (NOD-, LRR- and pyrin domain-containing protein 3) inflammasome. This macromolecular protein complex is involved in the pathogenesis of many cardiovascular diseases [55]. Thus, by increasing the production of ketone bodies, SGLT2 inhibitors may beneficially affect myocardial metabolism and show anti-inflammatory and anti-arrhythmic effects [56].

The ability of SGLT2 inhibitors to interact with the sodium-hydrogen exchanger NHE1 (Na^+^/H^+^ exchanger) may play an important role in myocardial remodeling in HF [57]. This exchanger, which is associated with the cell membrane, plays an important role in maintaining intracellular ionic homeostasis. In the failing heart, an increase in NHE1 protein expression is reported. It leads to increased intracellular sodium and calcium concentrations, inducing pro-oxidative and prothrombotic processes, which results in cardiomyocyte damage, HF, and even cardiovascular death [58,59]. By blocking the NHE1 exchanger in cardiomyocytes, SGLT2 inhibitors may counteract these processes and slow adverse myocardial remodeling [37]. It has also been shown that SGLT2 inhibitor therapy can inhibit myocardial fibrosis by blocking the TGF beta/Smad pathway, collagen synthesis, ∝-smooth muscle actin, connective tissue growth factor, extracellular matrix (ECM) remodeling, and matrix metalloproteinase 2 [60,61].

The effect of SGLT2 inhibitors on reducing the amount of epicardial adipose tissue and the beneficial impact of this phenomenon on fibrotic processes, improvement in myocardial contractility, prevention of arrhythmias, and HF seem to be very appealing [62,63].

### 4.4. Nephroprotective Effects of Gliflozins

The nephroprotective effect of SGLT2 inhibitors is related to the tubuloglomerular feedback, which is one of the mechanisms of glomerular filtration regulation [64]. In diabetes, SGLT2 receptors are overactivated. The reabsorption of glucose and sodium from the proximal tubule is increased. Low sodium concentration in the filtrate that reaches the macula densa leads to a local increase in adenosine concentration, which results in dilation of the afferent arteriole. This leads to hyperfiltration, increased intraglomerular pressure, and glomerular damage. By inhibiting SGLT2 receptors in the proximal convoluted tubule (PCT), SGLT2 inhibitors decrease glucose and sodium reabsorption, increase natriuresis and fluid loss, and reduce atrial natriuretic peptide secretion, causing constriction of the efferent arteriole. At the same time, increased sodium concentration in the glomerular filtrate activates vasoconstriction of the efferent arteriole and leads to the dilation of the efferent arteriole by inhibiting renin release. The above processes at the glomerular vascular level counteract hyperfiltration and show long-term protective effects on renal function [64,65]. Initiation of treatment with SGLT2 inhibitors may initially be associated with a slight decrease in GFR. However, these drugs slow the decrease in GFR in long-term follow-up [66]. A reduction in local inflammation and fibrosis also seems likely among other possible mechanisms of action of SGLT2 inhibitors responsible for the nephroprotective effect [67,68].

This protection of renal function is critical in patients with HF, as it prevents water intoxication and resistance to diuretics [69]. In addition, unlike classical diuretics, SGLT2 inhibitors reduce the extravascular fluid volume by minimally reducing intravascular fluid volume [7]. The reduction in the fluid volume and intravascular pressure activates the systemic and local renin-angiotensin-aldosterone system (RAAS). However, the activation occurs by angiotensin II type 2 receptor and not by type 1 receptor, which induces a protective effect on the cardiovascular system through vasodilation, sodium excretion, and anti-inflammatory effects, preventing hypertrophy and arrhythmias [70].

The nephroprotective effect of dapagliflozin was assessed by the DAPA-CKD trial, which included patients with chronic kidney disease (GFR 25–75 mL/min/1.73 m) regardless of diabetes [71]. The trial was terminated prematurely due to the benefits of dapagliflozin. It was shown that the use of dapagliflozin in patients with chronic kidney disease was associated with a significantly lower risk of a composite endpoint defined as a reduction in GFR > 50% or the occurrence of end-stage renal disease or death from renal and cardiovascular causes compared to the control group (9.2% vs. 14.5% HR, 0.61; 95% CI 0.51–0.72; *p* < 0.001, respectively). Treatment with dapagliflozin also significantly reduced overall mortality in this patient group (4.7% vs. 6.8% HR 0.69; 95% CI 0.53–0.88; *p* = 0.004, respectively) [72].

The nephroprotective effect of gliflozins was also confirmed in studies on the effectiveness of treatment of HFrEF. In the EMPEROR-Reduced trial, the use of empagliflozin was associated with a significantly slower decline in GFR compared to placebo (−0.55 vs. −2.28 mL/min/1.73 m^2^ per year, HR 1.73; 95% CI 1.1–2.37; *p* < 0.001). Additionally, the prespecified analysis showed a reduction in the composite endpoint defined as the implementation of renal replacement therapy, kidney transplantation, and a significant decrease in GFR (1.6% vs. 3.1%, HR 0.50; 95% CI 0.32–0.77). In the SOLOIST-WHF trial, patients on sotagliflozin with type 2 diabetes and HF had a reduction in adverse cardiovascular events. However, the use of sotagliflozin was not associated with a lower rate of GFR decline (0.34 vs. −0.18; *p* = 0.78). A similar result was obtained in the SCORED trial. The use of sotagliflozin in patients with chronic kidney disease (inclusion criteria: eGFR 25–60 mL/min/1.73 m^2^) and type 2 diabetes was associated with a significant reduction in adverse cardiovascular events. However, there was no beneficial effect of sotagliflozin on renal function, defined as a decrease in eGFR < 15 mL/min/1.73 m^2^ or <50% of the baseline value, need for dialysis or kidney transplantation (0.5% vs. 0.7%; *p* = 0.11) [73]. Both trials on sotagliflozin were prematurely terminated due to the discontinuation of funding. Therefore, a potentially beneficial effect of sotagliflozin on renal function in long-term follow-up cannot be excluded.

EMPA-REG OUTCOME and CANVAS PROGRAM trials showed a reduction in adverse events in patients with type 2 diabetes who used SGLT2 inhibitors. These adverse events included worsening of renal function such as increased albuminuria, decreased GFR, and the need for renal replacement therapy [8,16]. However, too few participants with chronic kidney disease prevented a reliable assessment of the effect of SGLT2 inhibitors on the progression of diabetic nephropathy. CREDENCE was the first trial to accurately assess the impact of SGLT2 inhibitors on the progression of chronic kidney disease in patients with type 2 diabetes [18]. Patients on canagliflozin had a 30% reduction in the risk of the primary composite endpoint, including the occurrence of end-stage renal disease (need for dialysis, kidney transplantation, or decrease in GFR < 15 mL/min/1.73 m^2^; HR 0.70; 95% CI 0.59–0.82; *p* = 0.00001), doubling of serum creatinine levels, or cardiovascular or kidney-related death. The risk of end-stage renal disease, including the doubling of creatinine levels or death from advanced kidney disease, was decreased by 34% (HR 0.66; 95% CI 0.53 to 0.81; *p* < 0.001). After introducing angiotensin II receptor blockers and angiotensin-converting enzyme inhibitors, canagliflozin was the first drug for the treatment of diabetic nephropathy, which significantly improved the prognosis in this group of patients [18,74].

### 4.5. Increased Hematocrit

Increased hematocrit is reported in patients treated with SGLT2 inhibitors [8]. This is usually associated with a decrease in total plasma volume found mainly in the initial phase of treatment and associated with a diuretic effect [75]. However, the EMPA-HEART CardioLink-6 trial showed that the use of SGLT2 inhibitors was additionally associated with increased erythropoietin, decreased ferritin, and decreased mean erythrocyte hemoglobin concentration (MCHC). These changes indicated that increased erythropoiesis was partially involved in changes in hematocrit [32]. In addition, studies showed that increased red blood cell count and hemoglobin concentrations were potent mediators of reduced HF hospitalizations and cardiovascular deaths when SGLT2 inhibitors were used [76,77]. These data may indicate that this group of drugs benefits due to an increase in hemoglobin concentration and red blood cell count, thereby increasing oxygen delivery to the myocardium. However, no differences were found in terms of the benefits of SGLT2 inhibitors between patients with ischemic and non-ischemic etiologies of HF [20].

In addition to stimulating the marrow to produce erythrocytes, erythropoietin has pleiotropic effects and may counteract apoptosis and protect the myocardium from reperfusion injury. Erythropoietin stimulates angiogenesis and has anti-inflammatory effects [78,79]. Another hypothesis is that increased erythrocyte count may merely be a biomarker of the impact of SGLT2 inhibitors on a common metabolic pathway responsible for cardioprotection and increased red blood cell production [80]. The use of SGLT2 inhibitors leads to glucosuria, which results in decreased glucose concentrations in tissues and activation of metabolic pathways associated with the starvation state. As a result, increased gluconeogenesis and ketogenesis are observed. At the molecular level, gluconeogenesis and ketogenesis are dependent on the activation of sirtuin 1 (SIRT1)-dependent intracellular pathways. Through deacetylation of many proteins, this enzyme affects the expression of genes responsible for maintaining cellular energy homeostasis. Activating signaling pathways associated with SIRT1 may lead to increased mitochondrial biogenesis, improved autophagy, and reduced oxidative stress in cardiomyocytes [81]. SIRT1 can also increase the activity of hypoxia-inducible factor 2 (HIF-2 alpha). This factor enhances the transcription of genes for erythropoietin, leading to increased erythropoiesis and elevated hematocrit [82].

## 5. Safety of Gliflozins

Some researchers paid attention to the adverse effects of SGLT2 inhibitors [83], of which genitourinary infections were the most prevalent. In the EMPA-REG and CANVAS trials, the number of infections caused by *Candida* species was 3–4 times higher than in the control group [8,16]. The risk factors for genitourinary infections during SGLT2 inhibitor use include female sex and past genitourinary infections. Of note, higher glycated hemoglobin levels were not associated with a higher incidence of the infection [84].

A high rate of treatment discontinuation with SGLT2 inhibitors was reported in patients who had an episode of genitourinary tract infection. Infection prophylaxis plays a significant role in the prevention of infections. Adequate intimate hygiene is crucial as it allows a considerable reduction in infections (40.8% in the control group vs. 4.8% in the group instructed about hygiene).

On the other hand, in the CANVAS trial, a significantly higher incidence of limb amputations was reported in patients treated with canagliflozin compared to the placebo group (6.3 vs. 3.37 per 1000 person-years, respectively), which could indicate increased limb ischemia [85]. In the whole study group, patients with a history of amputation and other typical risk factors for limb ischemia were most at risk for limb amputation. Additionally, the risk associated with canagliflozin was similar in all subgroups. No significant interactions were found between canagliflozin treatment and a history of peripheral arteriosclerosis, previous amputations, or the use of other drugs. In each subgroup, the number of amputations was lower than the predicted number of cardiovascular events that were avoided due to canagliflozin treatment [86].

However, this does not change the fact that the complication was serious. Other adverse effects include an increased risk of dehydration, severe hypoglycemia, or ketoacidosis [87]. Reports of euglycemic diabetic ketoacidosis (euDKA) seem to be of particular concern. One of the proposed mechanisms that explains the possibility of euDKA in patients treated with SGLT2 inhibitors is the inhibition of the SGLT2 cotransporter in pancreatic alpha cells, which may result in excessive glucagon secretion [88]. As a result, a change in the glucagon-insulin ratio and a glucosuric effect are reported, which can lead to an overproduction of ketone bodies and ketoacidosis due to a low-carbohydrate diet (which is another risk factor) [89]. DAPA-HF and EMPEROR-Reduced trials showed that none of the adverse events reached statistical significance compared to placebo. It was also related to renal adverse events, although patients with chronic kidney disease were enrolled in these trials [20,23].

## 6. SGLT2 Inhibitors and the ESC Guidelines

The 2021 ESC guidelines for the diagnosis and treatment of HF introduced significant changes to the treatment algorithm for HF with reduced LVEF. As a result, gliflozins received the highest recommendation (Class IA) with the highest level of evidence (A). The treatment algorithm for HFrEF is based on four main groups of drugs that modify the natural course of HF (i.e., ARNI/ACE inhibitors, beta-blockers, mineralocorticoid receptor blockers, and SGLT2 inhibitors).

Due to the different mechanisms of action of these drugs, their combined effect can change the natural course of HF by reducing the risk of hospitalization and death from HF. Previous guidelines recommended the introduction of drugs to improve patient prognosis. Currently, the four main groups of drugs should be used together as soon as possible. Dapagliflozin and empagliflozin are recommended regardless of the patient’s diabetes status. It is stressed that their diuretic effect may translate into a reduced need for loop diuretics. Gliflozin therapy may increase the risk of recurrent genital fungal infections, and a slight reversible decrease in GFR may be expected at the beginning of treatment. Therapy with SGLT2 inhibitors should be continued as they slow down the deterioration of renal function in the long-term follow-up.

In the most recent ESC HF guidelines, there is the concept of patient phenotyping, particularly in comorbidities. SGLT2 inhibitors such as canagliflozin, dapagliflozin, empagliflozin, ertugliflozin, and sotagliflozin are recommended (IA) for the prevention of HF and cardiovascular death and deterioration in renal function in patients with type 2 diabetes and cardiovascular disease and/or cardiovascular risk factors or chronic kidney disease. In addition, dapagliflozin, empagliflozin, and sotagliflozin are also recommended (IA) for patients with type 2 diabetes and HfrEF to reduce HF-related hospitalizations and cardiovascular death [90].

## 7. Future Directions

Due to the effectiveness of SGLT2 inhibitors in the treatment of heart failure and their pleiotropic mechanism of action, there are increasing attempts to use this group of drugs in other cardiovascular diseases. Preclinical studies on animal models of myocardial ischemia-reperfusion injury showed that the use of gliflozins could be associated with a reduced infarct size [91]. These data were confirmed by the SGLT2-I AMI PROTECT observational registry [92]. Patients with type 2 diabetes and the acute coronary syndrome who were on chronic SGLT2 inhibitors presented with significantly lower levels of inflammatory parameters and a smaller size of myocardial necrosis compared to patients who were not treated with gliflozins. The ongoing PRESTIGE-AMI trial will evaluate the effect of SGLT2 inhibitors administered periprocedurally at coronary intervention in the infarct area and the myocardial remodeling index [NCT04899479].

Another area of interest is related to the use of SGLT2 inhibitors to prevent arrhythmias and sudden cardiac death. One of the meta-analyses showed that SGLT2 inhibitors were associated with a significantly lower risk of atrial arrhythmias and a risk of sudden cardiac death in patients with type 2 diabetes and heart failure [93]. Different results were obtained by Sfairopoulos et al. in their meta-analysis. In patients with type 2 diabetes and/or heart failure and/or chronic kidney disease, gliflozin therapy was not associated with a lower risk of sudden cardiac death or ventricular arrhythmias compared with placebo. Neither low nor high doses of SGLT2 inhibitors seemed to have a positive effect on reducing the risk of sudden cardiac death [94]. Given the few ventricular arrhythmias, sudden cardiac deaths, and a wide range of confidence intervals in studies, more research is needed to assess the efficacy of gliflozins in preventing life-threatening arrhythmias. Several clinical trials are currently underway in this respect (NCT05174052, NCT04780438, NCT04117763, NCT04583813).

Gliflozins can also be used in the treatment of obesity. The use of SGLT2 inhibitors is associated with a weight loss of approximately 2–3 kg [95]. However, such a moderate weight reduction associated with compensatory mechanisms suggests that they are sufficiently effective in monotherapy. However, combination therapies with other agents used to treat obesity are more commonly postulated. Due to their different mechanism of action, gliflozins can be used with GLP-1 receptor analogs. By inducing glucosuria, SGLT2 inhibitors can counteract the reduction in energy expenditure at the time of using appetite suppressants [96]. However, additional studies are warranted to accurately assess the efficacy of gliflozins in the treatment of obesity.

## 8. Conclusions

Multidirectional effects of SGLT2 inhibitors on the cardiovascular system have proven to be highly beneficial, although their exact mechanism of action is still unclear. SGLT2 inhibitors effectively prevent hospitalizations, and dapagliflozin has also been shown to reduce overall and cardiovascular mortality in patients with HFrEF regardless of the coexistence of type 2 diabetes. The results of previous studies formed the basis for changes in the treatment of patients with HfrEF, which was reflected in the 2021 ACC/AHA recommendations and current recommendations of the European Society of Cardiology [90,97]. These drugs have become another mainstay of treatment for patients with HfrEF. All patients with HfrEF and GFR > 30 mL/min/1.73 m^2^ should be treated with an SGLT2 inhibitor, regardless of coexisting diabetes. If the favorable results of the EMPEROR-Preserved trial are confirmed in the ongoing DELIVER trial (NCT03619213), gliflozins may become a mainstay in the treatment of chronic HfpEF and HfmrEF.

## Figures and Tables

**Table 1:** The results of main trials on the cardiovascular safety of SGLT2 inhibitors in patients with type 2 diabetes [8,15,16].

	EMPA-REG OUTCOME	CANVAS PROGRAM	DECLARE-TIMI 58
Number of patients with type 2 diabetes	7020 (with cardiovascular disease)	10,141 (with cardiovascular disease 65%, or ≥two risk factors)	17,160 (with cardiovascular disease or many risk factors)
Drug	empagliflozin	canagliflozin	dapagliflozin
Primary endpoint	death from cardiovascular causes, non-fatal myocardial infarction, or non-fatal stroke	death from cardiovascular causes, non-fatal myocardial infarction, or non-fatal stroke	death from cardiovascular causes, myocardial infarction, ischemic stroke
Results	Reduction by 14% (HR 0.86 95% CI 0.74–0.99 *p* = 0.04)	Reduction by 14% (HR 0.86 95% CI 0.75–0.97 *p* = 0.02)	Reduction by 7% (HR 0.93 95% CI 0.84–1.03 *p* = 0.17)
Adverse event	Hospitalization for heart failure	Hospitalization for heart failure	Hospitalization for heart failure
Results	Reduction by 35% (HR 0.65 95% CI 0.5–0.85; *p* = 0.002	Reduction by 33% (HR 0.67 95% CI 0.52–0.87; *p* = 0.002)	Reduction by 27% (HR 0.73 95% CI 0.61–0.88)

**Table 2:** Characteristics of patients participating in the trials related to the efficacy of SGLT2 inhibitors in the treatment of heart failure with reduced ejection fraction [20,23,24].

	DAPA-HF	EMPEROR-Reduced	SOLOIST-WHF
Drug	dapagliflozin	empagliflozin	sotagliflozin
HFrEF (%)	100	100	79
NYHA III and IV (%)	32	25	50
Type 2 diabetes (%)	42	50	100
(median) NT-proBNP pg/mL	1437	1907	1780
GFR < 60mL/min/1.73 m^2^ (%)	41	48	70
ARNIs (%)	11	20	17

## Data Availability

Not applicable.
